# Spontaneous Ventilation Combined with Double-Lumen Tube Intubation during Thoracic Surgery: A New Anesthesiologic Method Based on 141 Cases over Three Years

**DOI:** 10.3390/jcm12206457

**Published:** 2023-10-11

**Authors:** Zsolt Szabo, Csongor Fabo, Matyas Szarvas, Maria Matuz, Adam Oszlanyi, Attila Farkas, Dora Paroczai, Judit Lantos, Jozsef Furak

**Affiliations:** 1 Doctoral School of Multidisciplinary Medicine, University of Szeged, H-6720 Szeged, Hungary; 2 Department of Anesthesiology and Intensive Therapy, University of Szeged, H-6720 Szeged, Hungary; 3 Institute of Clinical Pharmacy, Faculty of Pharmacy, University of Szeged, H-6720 Szeged, Hungary; 4 Department of Anesthesiology and Intensive Therapy, Bács-Kiskun County Teaching Hospital, H-6000 Kecskemét, Hungary; 5 Department of Thoracic Surgery, Markusovszky University Teaching Hospital, H-9700 Szombathely, Hungary; 6 Department of Medical Microbiology, University of Szeged, H-6720 Szeged, Hungary; 7 Department of Neurology, Bács-Kiskun County Teaching Hospital, H-6000 Kecskemét, Hungary; 8 Department of Surgery, University of Szeged, H-6720 Szeged, Hungary

**Keywords:** non-intubated thoracic surgery, VATS, spontaneous breathing, mechanical ventilation, SVI

## Abstract

Background: Non-intubated thoracic surgery has not achieved widespread acceptance despite its potential to improve postoperative outcomes. To ensure airway safety, our institute has developed a technique combining spontaneous ventilation with double-lumen tube intubation (SVI). This study aimed to verify the feasibility and limitations of this SVI technique. Methods: For the SVI method, anesthesia induction involves fentanyl and propofol target-controlled infusion, with mivacurium administration. Bispectral index monitoring was used to ensure the optimal depth of anesthesia. Short-term muscle relaxation facilitated double-lumen tube intubation and early surgical steps. Chest opening preceded local infiltration, followed by a vagal nerve blockade to prevent the cough reflex and a paravertebral blockade for pain relief. Subsequently, the muscle relaxant was ceased. The patient underwent spontaneous breathing without coughing during surgical manipulation. Results: Between 10 March 2020 and 28 October 2022, 141 SVI surgeries were performed. Spontaneous respiration with positive end-expiratory pressure was sufficient in 65.96% (93/141) of cases, whereas 31.21% (44/141) required pressure support ventilation. Only 2.84% (4/141) of cases reversed to conventional anesthetic management, owing to technical or surgical difficulties. Results of the 141 cases: The mean maximal carbon dioxide pressure was 59.01 (34.4–92.9) mmHg, and the mean lowest oxygen saturation was 93.96% (81–100%). The mean one-lung, mechanical and spontaneous one-lung ventilation time was 74.88 (20–140), 17.55 (0–115) and 57.73 (0–130) min, respectively. Conclusions: Spontaneous ventilation with double-lumen tube intubation is safe and feasible for thoracic surgery. The mechanical one-lung ventilation time was reduced by 76.5%, and the rate of anesthetic conversion to relaxation was low (2.8%).

## 1. Introduction

Thoracic surgery and anesthesia have undergone a paradigm shift over the last few decades, and specialists have focused on a minimally invasive approach. Video-assisted thoracic surgery (VATS) is the gold standard for minor and major pulmonary resections and thymectomies [1,2,3,4]. Traditionally, general anesthesia, muscle relaxation and mechanical one-lung ventilation (OLV) are mandatory for these procedures. However, complications and adverse effects, such as alveolar damage (endothelial glycocalyx damage, capillary shear stress and edema formation), residual neuromuscular blockades and postoperative nausea and vomiting due to mechanical OLV commonly occurred in a time-dependent manner [5,6,7,8,9]. Spontaneous ventilation has been shown to have immunological benefits (by reducing the negative effects on natural killer cells and lymphocytes) [10,11]. Initially, spontaneous ventilation and VATS procedures were combined in non-intubated thoracoscopic surgeries (NITS), which resulted in fewer complications and lower potential stress [12,13]. The main concern with the non-intubated method was airway safety. Anesthetists have a limited window of opportunity for securing the airway and may encounter difficulties in tracheal intubation in the lateral position. In emergency situations, conversion from one technique to another can be difficult and time-consuming. With the non-intubated technique, minimal invasiveness and airway safety must be balanced according to the concept recommended by the Enhanced Recovery After Surgery Society [14]. Double-lumen tube (DLT) placement is necessary to ensure maximal airway safety. However, the endotracheal tube may act as a foreign body and activate the cough reflex. Cough reflex elimination with a vagal nerve blockade is commonly used in non-intubated thoracic surgery. However, the combination of spontaneous breathing and double-lumen tube intubation is ’terra incognita’ in thoracic anesthesia. Our working group developed a new technique by merging the conventional and non-intubated techniques, namely spontaneous ventilation combined with double-lumen tube intubation (SVI). The advantage of SVI is that it has minimal invasiveness, providing the physiological breathing pattern of the spontaneous ventilation of the non-intubated method and ensuring safety similar to that of the classic technique. This study aimed to verify the feasibility and limitations of the SVI technique in 141 patients.

## 2. Materials and Methods

### 2.1. Study Design and Patient Selection

As a spontaneous breathing anesthetic approach, the SVI method is a novel alternative to previously reported techniques. In our case series, we applied the SVI method in a prospective, nonconsequential manner when patients met the inclusion and exclusion criteria (Table 1), and a dedicated anesthetist was assigned. Retrospective data including the intraoperative and early postoperative periods were collected and statistically analyzed to assess the feasibility of the SVI method as a primary endpoint and to identify any potential limiting factors. All patients were informed about the risks and benefits of the SVI method compared to classic anesthetic management before the operation.

This study was approved by the Ethical Committee of the Human Investigation Review Board at the University of Szeged (permission no.:4703/2020.01.20, Chairperson Professor Tibor Wittman, address: Koranyi fasor 8–10, Szeged, Hungary).

For patient selection, we applied our previously published criteria for non-intubated thoracic procedures (NITS) (Table 1) [15]. However, the contraindications for NITS do not exclude the use of the SVI method [16]. In our daily clinical setting, patients with a body mass index (BMI) < 34 without other contraindications were deemed suitable for SVI.

Preoperative pulmonological and anesthesiologic examinations were the same as those in the normal or NITS cases. From a surgical perspective, we included all cases of SVI that would also be suitable for normal VATS according to consensus meeting recommendations. We included patients with no advanced lung cancer (<7 cm, N0, or N1) [17].

Between 10 March 2020 and 28 October 2022, 141 surgeries were performed by our thoracic surgery team using the SVI approach for general anesthesia. All surgeries were performed by a single surgeon, and the patients were anesthetized by three anesthetists using the same procedural algorithm. Initially, we intended to perform 144 SVI procedures. Three cases were excluded from statistical analyses because spontaneous breathing did not return by the end of the surgery.

### 2.2. Anesthetic Management

Three-lead ECG, oxygen saturation (SpO_2_) and invasive blood pressure measurements were performed. The depth of anesthesia was monitored using the bispectral index (BIS, Medtronic Vista). Anesthesia was induced with fentanyl (1–1.5 µg/kg) and propofol using target-controlled infusion (Schnider model) with effect-site targeting. Considering the induction effect, the site target concentration was generally set between 4 and 6 µg/mL, depending on individual patient characteristics. Subsequently, the target concentration was modified to keep the BIS value between 40 and 60.

Mivacurium chloride (0.1–0.15 µg/kg), a short-acting non-depolarizing muscle relaxant, was used to ensure optimal conditions for intubation. Although the dose of mivacurium that was used was below the recommended dose for intubation, we found that, 180 s after drug administration, the conditions for intubation were good or excellent.

Similar to the gold standard approach, fiberoptic equipment (aScope, Ambu, Ballerup, Denmark) was used to confirm the proper position of the DLT. Confirmation of the proper tube position was crucial because further manipulation of the tube after the effects of the muscle relaxant diminished would not be well tolerated.

Muscle relaxation facilitated the DLT insertion and helped in early surgical steps. After the thoracic cavity was opened, a paravertebral nerve blockade for pain relief and a vagal blockade to prevent the cough reflex were performed. Spontaneous breathing returned after the muscle relaxant effect was eliminated. In unexpected cases, when spontaneous breathing was unsatisfactory (low tidal volumes, bradypnea), temporary pressure support ventilation with a low-flow trigger (1.0 L/min) was used until the muscle relaxant effect was fully eliminated.

Intraoperatively, altering the FiO_2_ (40–100%) and applying 3–5 H_2_O cm positive end-expiratory pressure (PEEP) to the dependent lung helped keep the SpO_2_ and PaCO_2_ within normal or close-to-normal ranges. Severe hypercapnia or hypoxia was also prevented or managed by applying pressure support ventilation to the dependent lung.

In case of necessity, the intraoperative evaluation of air leakage involved conducting a water submersion test (WST). After filling the thoracic cavity with saline, manual positive pressure ventilation was synchronized with the patient’s spontaneous breathing activity. Furthermore, if it was necessary, we were able to apply pressure support ventilation to perform the leak test.

### 2.3. Rescue Maneuvers

#### 2.3.1. Hypotension

According to our intraoperative hemodynamic management protocol, when the mean arterial pressure was <60 mmHg, the systolic blood pressure was <90 mmHg or decreased by more than 25%, and ephedrin (5–10 mg) or phenylephrine (50–100 µg) was administered in divided doses.

#### 2.3.2. Hypoxia/Hypercapnia

**Figure 1:**
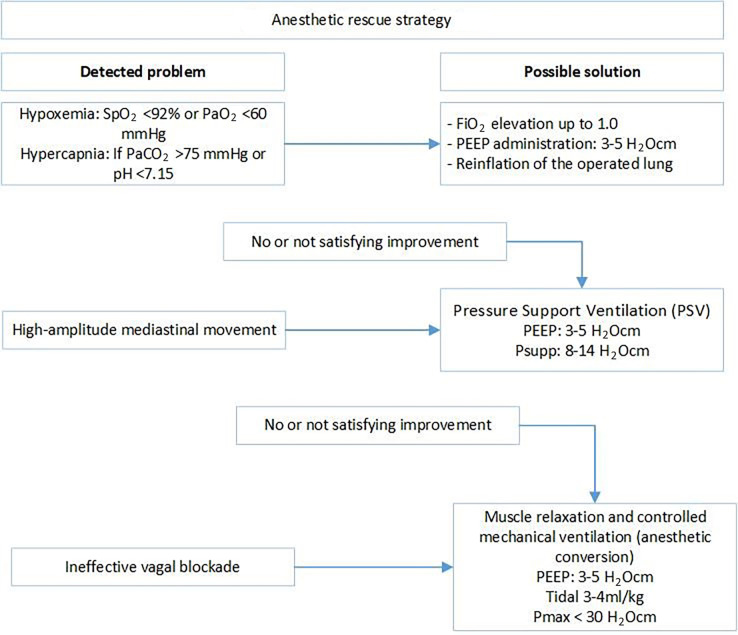
Anesthetic rescue strategy.

In patients with SpO_2_ < 92% or PaO_2_ < 60 mmHg, 3–5 H_2_O cm PEEP administration and FiO_2_ alteration were used to increase oxygenation. In the cases of PaCO_2_ > 75 mmHg or pH < 7.15, the non-dependent lung was considered to be re-inflated for a short period to eliminate carbon dioxide and improve oxygenation. The effect of reinflation is partial and temporary. Thus, if the improvement was not satisfactory, or as an initial step, we administered pressure support ventilation with 3–5 H_2_O cm PEEP and 8–14 H_2_O cm pressure support to assist in gas exchange. If hypercapnia and/or hypoxia were persistent, anesthetic conversion with muscle relaxation (0.05–0.1 µg/kg of mivacurium) and volume-controlled mechanical ventilation (PEEP: 3–5 H_2_O cm, tidal volume: 3–4 mL/kg, Pmax < 30 H_2_O cm) were applied (Figure 1).

#### 2.3.3. Technical Difficulties

Paradoxical mediastinal shifting is an accompanying phenomenon of spontaneous ventilation surgery. The mediastinum moves downward during inspiration and vice versa during expiration. If it was intolerable, pressure support ventilation was applied to overcome the issue. An ineffective vagal nerve blockade may result in coughing. If repeated vagal nerve infiltration was not feasible, anesthetic conversion was applied (Figure 1).

### 2.4. Regional Anesthetic Techniques

In our study, all regional anesthetic techniques were performed by the surgeon under direct vision to decrease the risk of complications associated with a regional blockade and to reduce the length of stay in the operating room. During VATS with SVI in routine cases, 5 mg/kg of lidocaine (2%) was administered at the site of incision at the fifth intercostal space in the mid-axillary line. After opening the thoracic cavity under thoracoscopic guidance, vagal and paravertebral blocks were performed. For the vagal nerve blockade, 3–5 mL of bupivacaine (0.5%) was administered close to the nerve (aortopulmonary window, left side; upper mediastinum, right side). A deep intercostal or paravertebral blockade was achieved by administering 4–5 mL of bupivacaine (0.5%) close to each intercostal nerve (from the second to the fifth intercostal space). The maximum amount of bupivacaine used was 0.5 mL/kg (2.5 mg/kg). In cases of open SVI, an intercostal nerve blockade was guaranteed by administering 4–5 mL of bupivacaine (0.5%) between the third and sixth intercostal spaces.

### 2.5. Surgical Technique

We performed the same VATS uniportal method during the SVI procedures that we published in our NITS study [15,18], with indications based on the European Society of Thoracic Surgeons consensus report [19] and the recommendation of the NITS pioneers [20,21].

### 2.6. Postoperative Care

Every patient was observed in the post-anesthesia care unit (PACU) for at least 2 h or until they met the criteria for leaving the PACU (visual analog scale [VAS] score < 3, Aldrete score > 9). Oxygen was administered to all patients via a face mask with 4–6 L/min O_2_ postoperatively to achieve a SpO_2_ of >94% or >88% in patients with chronic obstructive pulmonary disease. None of the patients required a higher level of oxygen or a higher degree of respiratory support (non-invasive ventilation) during the PACU stay or in the later postoperative period. None of the patients experienced fever or required bronchial secretion removal. Postoperatively, chest radiography was performed before and after the chest tube removal. Any pneumothorax, atelectasia/dystelectasia, infiltration or pleural fluid observed in the radiography were considered abnormal findings. The pain intensity during hospitalization was assessed using a numeric pain rating scale (NPRS). An NPRS > 3 was the intervention point, and a minor analgesic agent prescribed by the anesthetist was administered orally as soon as possible. For patients who underwent thoracotomy, a pleural catheter was inserted for continuous local anesthetic (0.1 mL/h/kgbw bupivacain 0.33%) administration.

### 2.7. Arterial Blood Sampling

**Figure 2:**
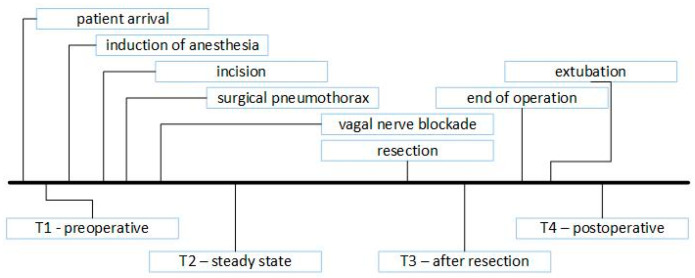
Timeline of blood sampling.

In cases of major pulmonary resection, blood samples were collected four times (T1, T2, T3 and T4; Figure 2). For T1, preoperative blood samples were collected before anesthesia induction, with a FiO_2_ of 0.21. For T2, steady-state blood samples were collected 15 min after the vagal nerve blockade. For T3, blood samples were collected 15 min after anatomical resection (and only during anatomical resections). For T4, postoperative blood samples were collected 30 min after the patient arrived in the recovery room and at a FiO_2_ of 0.5.

### 2.8. Data Collection and Analyses

Data were retrospectively collected from our medical system (e-MedSolution) and personal patient documentation. Personal patient data were also collected. Descriptive statistics were performed using R statistical software, version 4.2.2 (R Foundation, Vienna, Austria), and SPSS for Windows, version 26.0 (IBM Corp., Armonk, NY, USA). Descriptive statistics are presented as the mean ± standard deviation (SD) for continuous variables and as the count and percentage for categorical variables.

## 3. Results

### 3.1. Patient Characteristics

A total of 67 (47.52%) of the patients were men, and 74 (52.48%) were female patients. The mean age was 62.13 years (19–83), with a mean BMI of 25.82 (15.79–38.54) (Table 2). A total of 13 patients (9.22%) had previously undergone thoracic surgery. The surgical procedures were mainly lung resections (76 lobectomies, 22 segmentectomies, 25 wedge resections and 5 other procedures) and 13 thymectomies (Table 3).

### 3.2. Anesthetic Results

In 93 patients (93/141, 65.96%), spontaneous respiration, with or without 3–5 H_2_O cm PEEP administration, produced satisfactory gas exchange. In 44 cases (44/141, 31.21%), temporary or permanent PSV administration was necessary for supportive oxygenation and carbon dioxide removal. In four cases (4/141, 2.84%), repeated muscle relaxation and a return to a conventional anesthetic pathway were necessary. In one patient, high-amplitude mediastinal movement was intolerable despite the provision of applied pressure support ventilation. In two cases, vagal blockade ineffectiveness resulted in coughing under surgical manipulation, and muscle relaxation was necessary. In one patient, endotracheal tube malposition was confirmed, and anesthetic conversion was necessary for correction (Table 4).

We compared the possibly relevant factors influencing the necessity of pressure support ventilation during an SVI procedure (Table 5). The mean BMI was 26.9 (18.75–37.81) and 25.39 (15.79–38.54) in the PSV and in non-PSV groups, respectively. The incidence of asthma or COPD in the PSV group was 31.48% (14/44), and in the non-PSV group, it was 24.73% (23/93). According to the limited respiratory test parameter availability, the mean FEV_1_ and DLCO values were lower in the PSV group (75.71% ± 22.75% and 65.89% ± 22.04%) than in the non-PSV group (83.77% ± 18.35% and 76.55% ± 16.04%). In the PSV group, thymectomy was performed in five cases (5/44, 11.36%), in which pressure support ventilation was necessary due to a bilateral surgical pneumothorax.

After anesthetic induction and 5–10 min after the vagal nerve blockade, hypotension was common. Of the 141 patients, 65 (46.1%) required phenylephrine or ephedrine due to hypotension. Ephedrin or phenylephrin administration was necessary in 49 cases (49/95, 51.58%) in patients with hypertension or other cardiovascular diseases (CV group), and it was administered for 15 patients (15/44, 34.09%) without any cardiovascular disease (non-CV group). The systolic blood pressure reduction was 33.45 mmHg ± 18.71 mmHg in the CV group, and it was 28.67 mmHg ± 19.55 mmHg in the non-CV group. Moreover, the diastolic blood pressure reduction was 17.64 mmHg ± 10.68 mmHg and 15.49 mmHg ± 11.55 mmHg in the CV and non-CV groups, respectively (Table 6). However, none of our patients required continuous pharmacological hemodynamic support.

The mean one-lung ventilation time was 74.88 min (20–140 min). The mean mechanical and spontaneous OLV times were 17.55 min (0–115 min) and 57.73 min (0–100 min), respectively. The mechanical OLV time was reduced by 76.5%. The respiratory rate was altered by between 4 and 36 min. The mean minimum respiratory rate was 12.19 (4–30), whereas the mean maximum respiratory rate was 19.19 (6–36) (Table 7).

### 3.3. Blood Gas Results

At least one blood gas test result was available for 94 patients. The demographic comparison between the patient populations is shown in Table S1 in the Supplementary Materials.

**Figure 3:**
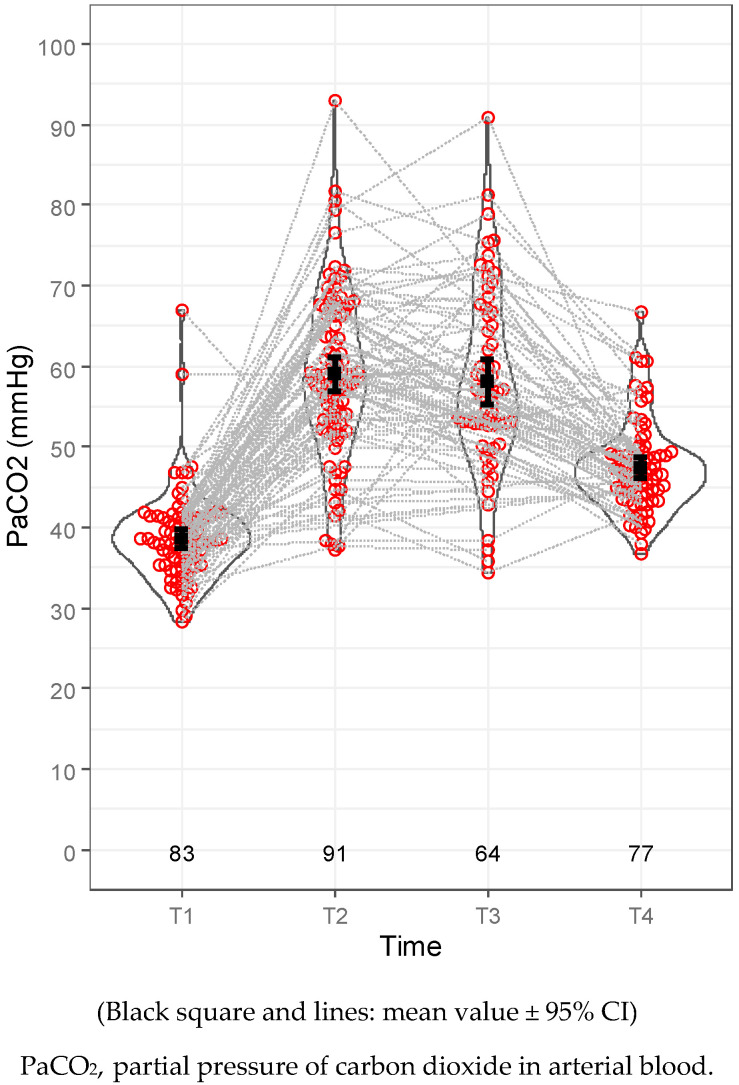
PaCO_2_ levels.

**Figure 4:**
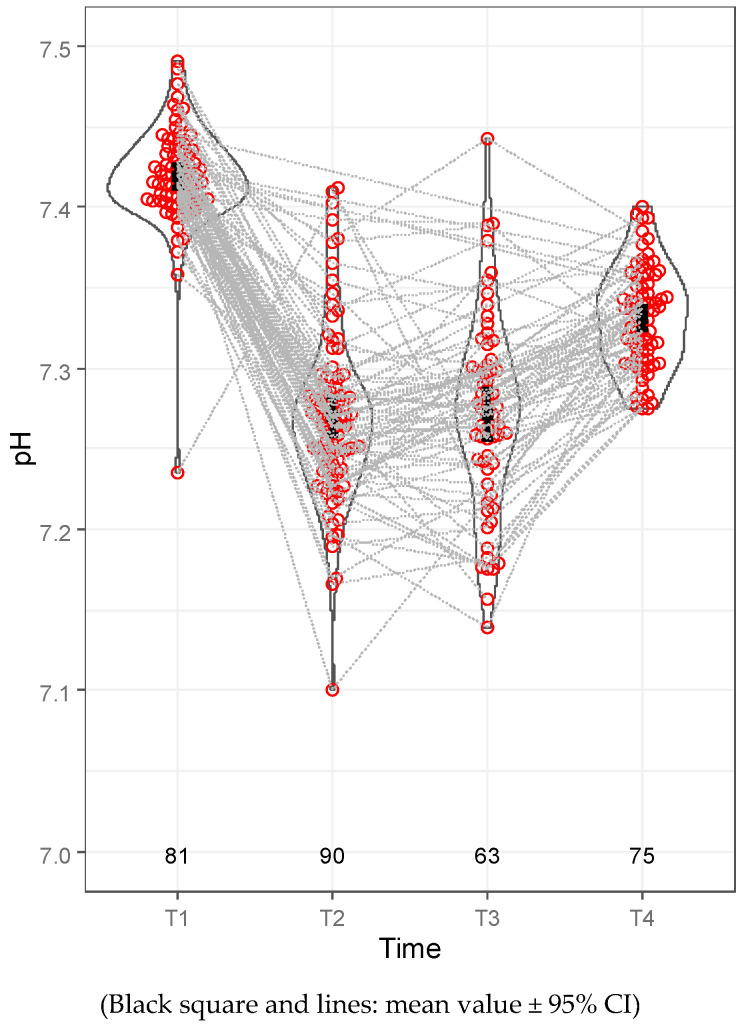
pH levels.

According to the blood gas results, the mean PaO_2_ level at time T2 was 115.97 mmHg (50.4–472.6 mmHg) and 143.831 mmHg (59.9–425.6 mmHg) at T3 (Table 8), and it was associated with a 93.96% (81–100%) mean minimal intraoperative oxygen saturation (Table 7). Hypercapnia, with or without respiratory acidosis, was a common but transient intraoperative complication. The mean PaCO_2_ level at T2 was 59.05 mmHg (37.1–92.9 mmHg), with a mean pH of 7.27 (7.1–7.41). The mean PaCO_2_ level at T3 was 58.17 mmHg (34.4–90.9 mmHg), accompanied by a mean pH of 7.27 (7.14–7.44). Hypercapnia and the acid–base discrepancy diminished in the early postoperative period. The mean PaCO_2_ level at T4 was 47.44 mmHg (36.7–66.7 mmHg) (Table 8, Figure 3). Consequently, the mean pH was 7.332 (7.275–7.401) (Table 8, Figure 4). The mean intraoperative lactate level was 0.701 mmol/L (0.22–1.86 mmol/L) at T2 and 0.667 mmol/L (0.22–1.83 mmol/L) at T3.

**Figure 5:**
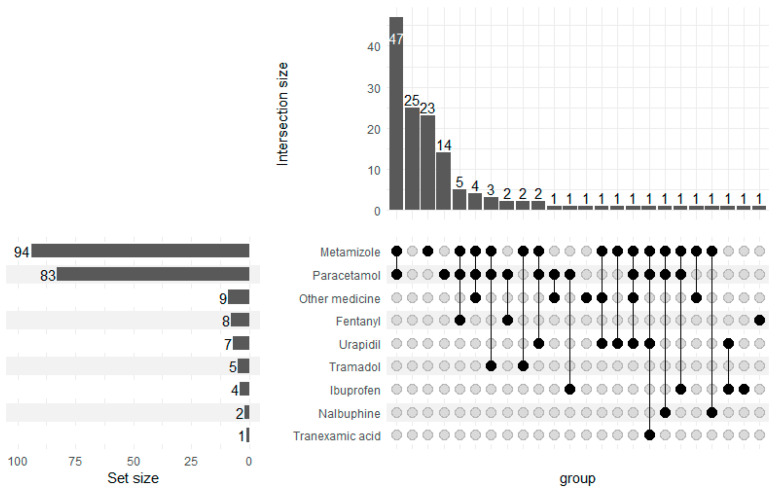
Post anesthesia care unit drug administration.

Upon completion of the surgery, all patients were extubated. After a mean of 68.18 min (30–170 min) of observation in the recovery room, all patients were transported back to the thoracic surgical ward. None of the patients required a higher flow of oxygen therapy, non-invasive ventilation, reintubation or intensive care unit admission. In the absence of contraindications, our patients routinely received 75 mg of diclofenac in the operating room as part of our multimodal pain management strategy. In the PACU, 25 of 141 patients did not require further analgesics. Metamizole (1 g, IV) and paracetamol (1 g, IV) were the most commonly administered analgesics postoperatively. A total of 94 (94/141, 66.67%) patients received metamizole, and 83 of the 141 patients (58.87%) received paracetamol. In half of the patients, when metamizol was necessary, it was co-administered with paracetamol (47/141, 33.33%). Approximately, 10–75 µg of fentanyl administration was necessary in 8 (5.67%) patients to achieve a VAS score <4. Tranexamic acid administration was necessary in 1 patient (1/141, 0.71%) because of the high rate of bleeding drained from the chest tube (Figure 5).

### 3.4. Surgical Intraoperative Results

A total of 3 of the 141 surgeries were intended for open SVI procedures. In 12 additional cases (12/138, 8.70%), conversion to thoracotomy was necessary, without anesthetic conversion (Table 9). Of these, 5 were due to oncological reasons (5/12, 41.67%), and 5 were due to technical difficulties (5/12, 41.67%). There was also bleeding in 2 cases (2/12, 16.67%). Overall, we performed 15 open thoracotomies (15/141, 10.64%) and 126 VATS (126/141, 89.36%) surgeries. The mean operation time was 80.6 min (25–150 min) and 80.2 min (25–150 min) in all cases and in cases without anesthetic conversion, respectively. The mean length of hospital stay was found to be 4.8 days (1–26 days) in all cases.

## 4. Discussion

The main concern with the non-intubated technique is airway safety. Without lung isolation, CO_2_ rebreathing (the pendelluft phenomenon) is a possible consequence of chest opening. Hypoxia and hypercapnia can develop as a result of pendelluft and paradoxical mediastinal shifting, which are the main obstacles to the widespread acceptance of NITS among anesthetists and surgeons [22,23,24,25]. In our practice, as published earlier by our workgroup [15], the non-intubated anesthetic approach encompasses BIS-guided propofol TCI with fentanyl administration for the induction of anesthesia, followed by laryngeal mask insertion. Therefore, the most relevant difference between SVI and NITS from our perspective is the question of airway safety. However, other workgroups (and their patients) may profit from general anesthesia itself.

SVI combines the beneficial effects of spontaneous ventilation thoracic procedures with airway safety via tracheal intubation using a double-lumen tube. The essential components of SVI are double-lumen tube intubation for maximal airway safety, short-acting non-depolarizing muscle relaxants for the early recovery of spontaneous ventilation, paravertebral blockades as part of multimodal pain management and vagal nerve blockades to prevent the cough reflex. Infiltration of the vagal nerve with 2–5 mL of bupivacain (0.25–0.5%) into the thoracic cavity is a widely accepted method [2,12,15,26,27], and repeated vagal nerve blockades may be necessary [26]. However, in our practice, a single infiltration was sufficient for a mean of 80.2 min (25–150 min). From the perspective of the anesthetist, the initial stage of SVI resembles that of conventional thoracic procedures. After cessation of the muscle relaxant, patients breathed spontaneously without coughing. In three cases, spontaneous breathing was not detected until the end of surgery, despite the reduced dose of mivacurim, although all patients underwent short procedures. Furthermore, anesthetic management was similar to that of the NITS procedures. From the surgeon’s perspective, there was no major difference between SVI and NITS.

Double-lumen endotracheal tube placement provides an opportunity for all anesthetic interventions (recruitment maneuvers and fiberoptic control) with complete lung isolation. Therefore, if a surgical or anesthetic complication occurs, conversion to the conventional anesthetic pathway is easy and safe. The most risky part of the NITS procedure is the conversion process under aggravated circumstances (2–11% of cases) [12,27,28,29,30,31], which is prevented. Although single-lumen intubation with a blocker may be considered less invasive, a bronchial blocker is used as a secondary option in our institute for situations in which the placement of the DLT is not possible. When explained with the potential advantages, DLTs offer greater stability and are less prone to dislodgment during surgery, as well as more effective lung isolation. Furthermore, it is essential to consider the cost-effectiveness of each approach and the available resources.

Furthermore, the incidence of unforeseen difficult airways and difficult intubation can reach up to 20%. According to Langiano et al., the overall incidence of difficult airways is 16% in the thoracic surgical patient population, whereas the frequency of unexpected difficult airways is 5.2% [32]. Corso et al. reported in a retrospective study of 763 patients that difficult intubation occurred in 13.6% of cases, challenging mask ventilation occurred in 9%, and a combination of both difficulties occurred in 2% [33]. In such instances, SVI may provide a secure procedure for spontaneous ventilation. Moreover, when the indication for conversion is surgical (thoracotomy), muscle relaxation and controlled mechanical ventilation are not required. Spontaneous ventilation surgeries require surgeons to leave their comfort zone because paradoxical mediastinal shifting and diaphragmatic movements create an unusual surgical field. In their meta-analysis, Shi et al. reported that mediastinal and diaphragmatic factors were the most common complications leading to anesthetic conversion (7% and 4%, respectively) [34]. In our SVI case series, muscle relaxation and permanently controlled mechanical OLV occurred in one patient due to intolerable mediastinal shifting (1/141, 0.71%). However, the incidence of disturbed mediastinal movement was higher but was managed with pressure support ventilation. Two of our anesthetic conversions (2/141, 1.42%) were due to ineffective vagal nerve blockades, and repeated vagal nerve infiltration was technically infeasible. The fourth anesthetic conversion was due to DLT dislodgement (1/141, 0.71%). The remaining 97.16% of patients did not require further muscle relaxation after the initial dose of the muscle relaxant for induction. Twelve surgical conversions from VATS SVI to open SVI were uneventful and did not require anesthetic conversion.

Gas exchange imbalance is common during spontaneous ventilation surgery. Hypoxia and hypercapnia have also been observed during NITS [34]. In SVI, hypoxia is easily compensated by a higher FiO_2_, PEEP administration or applying pressure support. However, it is important to emphasize that the peak airway pressure is lower during spontaneous ventilation, with or without pressure support, than that during controlled ventilation [16]. Hypercapnia is a multifactorial condition. However, deepening the anesthesia negatively affects respiratory activity. In contrast, mediastinal shifting reduces the tidal volume and lung compliance, and airway resistance is also increased by using a double-lumen tube. To overcome hypercapnia, pressure support ventilation is an intermediate step before anesthetic conversion to maintain spontaneous breathing. Hypercapnia and associated respiratory acidosis tend to be temporary, and the acid–base aberration is generally spontaneously corrected after the operated lung is reinflated [22,24,35,36]. We identified the lowest maximum breathing frequency of 6, which did not result in hypoxia or hypercapnia. This was attributed to the thymectomy performed, the patient’s overall good health, young age (36 years) and low BMI (19.1). According to our blood gas results taken 30 min after extubation (T4), the mean PaCO_2_ was 47.44 mmHg (36.7–66.7 mmHg), and the pH was 7.332 (7.275–7.401). However, hypercapnia itself may result in better ventilation–perfusion matching and reduce intraoperative lung injury by suppressing inflammatory responses [37,38]. Furák et al. reasoned that SVI is more physiological in relation to gas exchange, and the authors found that a significantly lower lowest oxygen saturation and higher maximum PaCO_2_ level were found in the non-intubated group (vs. the SVI group) [39].

In our SVI series, hypotension commonly occurred after anesthesia induction and vagal nerve blockades. In total, 46.1% of our patients needed temporary pharmacological hemodynamic support. For the rest of the patients (53.9%), reductions in blood pressure were below our hemodynamic management cut-off value, and thus, self-regulation was sufficient to normalize blood pressure. The incidence of hypotension and the extent of intraoperative blood pressure reduction were higher among patients in the cardiovascular disease (CV) group. The elevated occurrence of hypotension can be attributed to the medications routinely used by patients in the CV group. Among these patients, 52 (52/95, 54.74%) were taking beta-blockers, 75 (75/95, 78.95%) were on antihypertensive medications (ACE inhibitors, ARBs, Ca channel blockers, imidazoline/α-2 receptor agonists), 30 (30/75, 40%) were on combinations of multiple antihypertensive drugs, and 15 patients (15/95, 15.79%) were regularly taking antidiuretic agents. As described earlier, SVI shows better hemodynamic stability than that of the non-intubated technique [39]. The negative effects of controlled mechanical ventilation (CMV) have been extensively explored. Thus, the decrease in the mechanical OLV time (76.5%) suggests that our patients suffered from less oxidative stress, which may offer some immunological advantages [6,8,9,11].

Furák and Szabó previously published their results on SVI [16] and SVI lobectomies [39]. Their mean surgical time was 83.3 min (55–130 min) and 88.1 min (55–120 min), respectively. These times are similar to our mean surgical time of 80.2 min (25–150 min) and shorter than that reported in AlGhamdi’s study on non-intubated and intubated/relaxed VATS lobectomies (130.9, and 146.0 min) [40]. Furthermore, Moon et al., in their study of 115 non-intubated thoracoscopic surgeries, reported that the mean operation time was 130 min [31]. Similarly, Hung et al., in their study of 109 non-intubated thoracic procedures, reported a mean operative time of 124.4 min [41]. Although the focus of this study is not the detailed surgical results, by comparing Furak’s first SVI study with AlGhamdi’s study, it can be found that the mean postoperative length of hospital stay after VATS SVI lobectomies and open SVI lobectomies was shorter than the length of stay reported in AlGhamdi’s study after non-intubated and intubated/relaxed VATS lobectomies (3.7 and 4.8 days for SVI VATS/open lobectomies and 6.9 and 7.6 days for non-intubated and intubated/relaxed lobectomies) [16,40]. Our conversion rate from VATS to open thoracotomy was 8.7%. All conversions were due to technical oncological reasons or due to bleeding, which was not associated with the SVI method. According to Power et al.’s systematic review and meta-analysis, based on the results of 72.932 patients, the median conversion rate from VATS to thoracotomy for anatomical resections was 9.6% [42].

There are only a few established exclusion criteria for SVI, and patients indicated for VATS are also candidates for SVI. NITS has several exclusion criteria, such as potentially difficult airways or intubation, coagulation disorders or mental conditions, which are not exclusion reasons for SVI [7,40,43]. In our case series, the contraindications for SVI were a high BMI, patient refusal, elevated intracranial pressure, hemodynamic instability and right heart failure (Table 1). We accepted a higher threshold BMI as a general exclusion criterion for SVI (as well as for NITS) compared to other working groups because our national average BMI was slightly higher. Based on individual considerations, we sometimes made further concessions regarding the BMI if it was deemed justified by the patient’s condition and respiratory function.

### Limitations

Our study has several limitations. More detailed perioperative monitoring (respiratory function tests, blood gas analyses and laboratory tests) and postoperative outpatient follow-ups would provide more comprehensive physiological and pathophysiological data related to SVI. Having compared the conventional and non-intubated methods, our findings suggest that future investigations are required to further evaluate the advantages and disadvantages of the SVI method.

## 5. Conclusions

The maintenance of spontaneous respiration during thoracic procedures is a well-known anesthetic strategy [12,13,16,44,45]. The SVI method is safe and feasible for various thoracic procedures (VATS, open surgery, lung resection, thymectomy and diagnostic procedures) in appropriately selected patients. SVI has fewer exclusion criteria than NITS because of double-lumen tube intubation and a safe airway. Intubation makes conversion to the classic anesthetic management pathway easy, fast and comfortable and can facilitate all anesthesiologic procedures. The SVI method decreases mechanical OLV time by 76.5%. Thus, patients benefit from the advantageous effects of spontaneous breathing. Maintaining spontaneous ventilation diminishes potential airway risks (positive pressure ventilation and consequent alveolar damage) and preserves the function of the diaphragm (decreases the atelectatic zones in the dependent lung and may result in better ventilation–perfusion matching). It may also contribute to a lower incidence of postoperative complications (pneumonia, respiratory failure and residual neuromuscular blocking agent effect), a shorter length of hospital stay and a reduction in the duration of needing a chest tube. Further comparative assessments are required to determine the superiority of the SVI technique over NITS and other conventional anesthetic methods. Based on our findings, we suggest that all three approaches are applicable to thoracic anesthesia. The anesthetic method of choice is dependent on the individual needs of each patient.

## Figures and Tables

**Table 1:** Exclusion criteria of spontaneous breathing anesthetic techniques.

NITS	SVI
Patient refusal	Patient refusal
Mental disorder (lack of cooperability), elevated intracranial pressure	Elevated intracranial pressure
Sleep apnea syndrome	
Airway abnormalities, anticipated difficult airway	
BMI ≥ 34 kg/m^2^	BMI ≥ 34 kg/m^2^
Persistent cough or high airway secretion	
Elevated risk of regurgitation	
Coagulation abnormality, INR > 1.5	
Hemodynamic instability, right heart failure	Hemodynamic instability, right heart failure

NITS, non-intubated thoracic surgery; SVI, spontaneous ventilation combined with double-lumen tube intubation; BMI, body mass index; INR, International Normalized Ratio.

**Table 2:** Demographic parameters of SVI patients.

		All Cases	
		N = 141	100%
Sex	Male	67	47.52
	Female	74	52.48
Age	18–64	70	49.65
	65–	71	50.35
	mean ± SD	62.13 ± 13.56	
	min–max	19–83	
BMI	Underweight (BMI < 18.5)	6	4.26
	Normal weight (BMI: 18.5–24.9)	62	43.97
	Pre-obesity (BMI: 25–29.9)	49	34.75
	Obesity class I (BMI: 30–34.9)	19	13.48
	Obesity class II (BMI: 35–39.9)	5	3.55
	mean ± SD	25.82 ± 4.51	
	min–max	15.79–38.54	
ASA score	1	7	4.96
	2	95	67.38
	3	39	27.66
Smokers (current)	Yes	57	40.43
	No	84	59.57
Current or previous smokers	No	49	34.75
	Yes	89	63.12
	NA	3	2.13
Smoking intensity	Less than 20/day	30	
	More than 20/day	37	
	NA	22	
Smoking time	0–9 years	4	
	10–19 years	6	
	20–29 years	12	
	More than 30 years	44	
	NA	23	
Most relevant comorbidities	Hypertension	84	59.57
	Cardiovascular disease	41	29.08
	Asthma/chronic obstructive pulmonary disease	38	26.95
	Diabetes mellitus	27	19.15
	Previous thoracic surgery	13	9.22
Preoperative medications	Anti-hypertensive agent	75	53.19
	Rhythm/frequency control agent	54	38.30
	Anticoagulants, antiaggregants	44	31.21
	Other cardiovascular drugs, diuretics	18	12.77
	Pulmonological drugs	29	20.57
	Statins	24	17.02
	Antidiabetics (incl. insulin)	19	13.48
	Psychiatric drugs	28	19.86
	Other medications	34	24.11

BMI, body mass index; ASA score, American Society of Anesthesiology score.

**Table 3:** Surgical parameters of SVI patients n = 141.

	Mean	Range
Carlson Comorbidity Index	5.51	0–12
FEV_1_ (%) (n = 91)	82.45	22.3–126.4
DLCO (%) (n = 47)	73.67	35.3–106
Surgical time (min)	80.6	25–150
	**N**	**%**
Pneumonectomy	1	0.71%
Lobectomy	76	53.9%
Segmentectomy	22	15.6%
Wedge resection	25	17.73%
Pleural biopsy	2	1.42%
Exploration	2	1.42%
Thymectomy	13	9.22%

FEV_1_, forced expiratory volume in one second; DLCO, diffusing capacity of the lungs for carbon monoxide.

**Table 4:** Success of SVI and anesthetic conversions of SVI (n = 141).

Overall Success of SVI:	N	%
Spontaneous respiration with or without PEEP administration (non-PSV group)	93	65.96
Temporary/permanent pressure support ventilation (PSV group)	44	31.21
Anesthetic conversion (muscle relaxation, controlled positive pressure OLV)	4	2.84
**Reasons for anesthetic conversion:**	**N**	**%**
Intolerable mediastinal movement	1	0.71
Ineffective vagal blockade	2	1.42
DLT malposition	1	0.71

SVI, spontaneous ventilation combined with double-lumen tube intubation; PEEP, positive end-expiratory pressure; OLV, one-lung ventilation; DLT, double-lumen tube.

**Table 5:** Patient characteristics of the PSV and non-PSV groups.

		PSV Group	Non-PSV Group	*p*
		N = 44	N = 93	
Sex	Male	24 (54.55%)	40 (43.01%)	^1^ 0.271
	Female	20 (45.45%)	53 (56.99%)	
Age	18–64	21 (47.73%)	47 (50.54%)	^1^ 0.855
	65–	23 (52.27%)	46 (49.46%)	
	mean ± SD	62.59 ± 12.86	61.09 ± 15.55	^2^ 0.553
	min–max	19–80	26–83	
BMI	Underweight (BMI < 18.5)	0 (0%)	6 (6.45%)	^3^ -
	Normal weight (BMI: 18.5–24.9)	17 (38.64%)	43 (46.24%)	
	Pre-obesity (BMI: 25.0–29.9)	18 (40.91%)	29 (31.18%)	
	Obesity class I (BMI: 30.0–34.9)	8 (18.18%)	11 (11.83%)	
	Obesity class II (BMI: 35.0–39.9)	1 (2.27%)	4 (4.3%)	
	mean ± SD	26.90 ± 4.18	25.39 ± 4.62	^2^ 0.068
	min–max	18.75–37.81	15.79–38.54	
ASA score	1	2 (4.55%)	5 (5.38%)	^4^ 0.936
	2	29 (65.91%)	63 (67.74%)	
	3	13 (29.55%)	25 (26.88%)	
Smokers (current)	Yes	14 (31.82%)	40 (43.01%)	^1^ 0.262
	No	30 (68.18%)	53 (56.99%)	
Current or previous smokers	No	17 (38.64%)	32 (35.56%)	^1^ 0.849
	Yes	27 (61.36%)	58 (64.44%)	
	NA	0	3	-
Most relevant comorbidities	Hypertension	29 (65.91%)	53 (56.99%)	^1^ 0.355
	Cardiovascular disease	11 (25%)	29 (31.18%)	^1^ 0.547
	Asthma/COPD	14 (31.82%)	23 (24.73%)	^1^ 0.306
	Diabetes mellitus	11 (25%)	16 (17.2%)	^1^ 0.358
	Previous thoracic surgery	6 (13.64%)	7 (7.53%)	^1^ 0.349
Spirometry	FEV1 % N	26	61	^2^ 0.043
	FEV1 % mean ± SD	75.71 ± 22.75	83.77 ± 18.35	
	DLCO % N	11	34	^2^ 0.044
	DLCO % mean ± SD	65.89 ± 22.04	76.55 ± 16.04	
	FVCL % N	24	51	^2^ 0.253
	FVCL % mean ± SD	88.45 ± 20.5	91.36 ± 16.01	
	FEV1/FVC (%) N	22	37	^2^ 0.215
	FEV1/FVC (%) mean ± SD	69.69 ± 11.84	71.94 ± 9.68	
Thymectomy	Yes	5 (11.36%)	8 (8.6%)	^1^ 0.756
	No	39 (88.64%)	85 (91.4%)	

^1^ Fisher’s exact test; ^2^ *t*-test; ^3^ Does not meet the criteria for Pearson’s chi-squared test; ^4^ Pearson chi-squared. Normality was tested via visual interpretation (Q–Q plot). Continuous variables were tested via an independent samples *t*-test to compare differences between groups, whereas categorical variables were analyzed using Pearson’s chi-squared test and Fisher’s exact test to compare the proportions of groups. Four cases, in which muscle relaxation and conversion to the classic anesthetic method were necessary, have been excluded from the statistical analysis.

**Table 6:** Hemodynamic support parameters of SVI patients.

		CV Group	Non-CV Group	*p*
		N = 95	N = 44	
Ephedrin or Phenylephrin administration	Yes	49 (51.58%)	15 (34.09%)	^1^ 0.068
	No	46 (48.42%)	29 (65.91%)	
Ephedrin dosage (mg)	N	28	8	^2^ 0.774
	mean ± SD	17.5 ± 11.18	16.25 ± 9.16	
Phenylephrin dosage (µg)	N	28	7	^2^ 0.473
	mean ± SD	257.14 ± 168.17	207.14 ± 136.71	
RRsys difference (mmHg)	N	91	43	^2^ 0.176
	mean ± SD	33.45 ± 18.71	28.67 ± 19.55	
RRdias difference (mmHg)	N	91	43	^2^ 0.291
	mean ± SD	17.64 ± 10.68	15.49 ± 11.55	

^1^ Fisher’s exact test; ^2^ *t*-test; Normality was tested via visual interpretation (Q–Q plot). Continuous variables were tested via an independent samples *t*-test to compare differences between groups, whereas categorical variables were analyzed using Fisher’s exact test to compare the proportions of groups. RRsys, systolic blood pressure; RRdias, diastolic blood pressure. Two patients were excluded from the statistical analysis, as no data were available regardinig their cardiovascular disease status.

**Table 7:** Anesthesiologic parameters of SVI patients (n = 141).

	Mean	Median	Std. Deviation	Minimum	Maximum
HR min	64.84	65.00	12.432	39	90
HR max	84.91	83.00	14.314	52	130
Pre RRSys	126.93	125.00	22.199	80	180
Pre RRDias	74.37	70.00	14.108	38	120
Post RRSys	94.92	90.00	20.594	46	145
Post RRDias	57.25	60.00	12.784	26	94
OLV ideje	74.88	75.00	25.521	20	140
Mech. OLV	17.55	15.00	17.245	0	115
Sp. OLV	57.73	60.00	24.685	0	130
Sp. OLV/OLV (%)	76.539	80.952	19.714	0	100
SpO_2_ Min	93.96	94.00	4.060	81	100
SpO_2_ Max	99.18	100.00	1.254	94	100
Resp. R. Min	12.19	12.00	3.302	4	30
Resp. R. Max	19.19	18.00	4.659	6	36

HR, heart rate; Pre RRSys, systolic blood pressure before vagal blockade; Pre RRDias, diastolic blood pressure before vagal blockade; Post RRSys, systolic blood pressure after vagal blockade; Post RRDias, diastolic blood pressure after vagal blockade; Mech. OLV, mechanical one-lung ventilation; Sp. OLV, spontaneous one-lung ventilation. OLV, one-lung ventilation; SpO_2_, oxygen saturation; Resp. R, respiratory rate.

**Table 8:** Blood gas results.

	Time		N	Mean	SD	Minimum	Maximum
FiO_2_	1	preoperative	82	0.210	0.000	0.210	0.210
	2	steady state	89	0.821	0.200	0.500	1.000
	3	after resection	62	0.829	0.197	0.500	1.000
	4	postoperative	77	0.500	0.000	0.500	0.500
pH	1	preoperative	81	7.419	0.032	7.235	7.491
	2	steady state	90	7.270	0.054	7.100	7.412
	3	after resection	63	7.271	0.061	7.139	7.443
	4	postoperative	75	7.332	0.032	7.275	7.401
PaCO_2_ (mmHg)	1	preoperative	83	38.619	5.642	28.300	66.900
	2	steady state	91	59.053	10.299	37.100	92.900
	3	after resection	64	58.167	11.293	34.400	90.900
	4	postoperative	77	47.438	5.670	36.700	66.700
PaO_2_ (mmHg)	1	preoperative	81	79.459	11.519	57.900	130.700
	2	steady state	91	115.969	67.318	50.400	472.600
	3	after resection	64	143.831	78.665	59.900	425.600
	4	postoperative	77	149.543	55.581	48.600	262.100
Lactate (mmol/L)	1	preoperative	71	0.815	0.329	0.260	2.110
	2	steady state	72	0.701	0.322	0.220	1.860
	3	after resection	56	0.667	0.293	0.220	1.830
	4	postoperative	66	0.780	0.303	0.230	1.600

FiO_2_, fraction of inspired oxygen; pH, potential of hydrogen; PaCO_2_, partial pressure of carbon dioxide in arterial blood; PaO_2_, partial pressure of oxygen in arterial blood.

**Table 9:** Surgical conversions from VATS to open thoracotomy.

Reason for Surgical Conversion (n = 12)	N	%
Technical difficulties	5	41.67
Oncological consideration	5	41.67
Bleeding	2	16.67
**Type of Converted Procedures (n = 12)**	**N**	**%**
Atypical resection	1	8.34
Segmentectomy	2	16.67
Lobectomy (included 3 sleeve lobectomies)	9	75.00

## Data Availability

The data presented in this study are available on request from the corresponding author. The data are not publicly available due to ethical restrictions.

## Supplementary Materials

The following are available online at https://www.mdpi.com/article/10.3390/jcm12206457/s1: Table S1: Comparison of demographic parameters of the “blood gas” and “no blood gas” groups.
