# Supplementary material for: Spontaneous Ventilation Combined with Double-Lumen Tube Intubation during Thoracic Surgery: A New Anesthesiologic Method Based on 141 Cases over Three Years
Source: J Clin Med. 2023 Oct 11;12(20):6457. doi: 10.3390/jcm12206457 (PMC10607362; doi:10.3390/jcm12206457)
Supplement: Supplementary file 1 [file jcm-12-06457-s001.zip › jcm-2631123-supplementary.pdf]

Supplementary Materials

Table S1. Comparison of demographic parameters of the "blood gas" and "no blood gas" groups.

		**Blood gas N=94**	**No blood gas N=47**	p
**Sex**	Male	45 (47.87%)	22 (46.81%)	[1]0.524
	Female	49 (52.13%)	25 (53.19%)	
**Age**	18-64	43 (45.74%)	27 (57.45%)	[1]0.129
	65 -	51 (54.26%)	20 (42.55%)	
	mean ± SD	63.50 ± 13.42	59.38 ± 13.58	[2]0.089
	min-max	19-83	22- 80	
**BMI**	Underweight (BMI < 18,5)	2 (2.13%)	4 (8.51%)	[3]-
	Normal weight (BMI: 18,5 – 24,9)	44 (46.81%)	18 (38.3%)	
	Pre-obesity (BMI: 25 – 29,9)	37 (39.36%)	12 (25.53%)	
	Obesity class I (BMI: 30 – 34,9)	8 (8.51%)	11 (23.4%)	
	Obesity class II (BMI: 35 – 39,9)	3 (3.19%)	2 (4.26%)	
	mean ± SD	25.76 ± 4.21	25.94 ± 5.12	[2]0.829
	min-max	18.00 - 38.54	15.79 - 35.34	
**ASA score**	1	2 (2.13%)	5 (10.64%)	[4]0.086
	2	66 (70.21%)	29 (61.7%)	
	3	26 (27.66%)	13 (27.66%)	
**Smokers (current)**	Yes	33 (35.11%)	24 (51.06%)	[1]0.101
	No	61 (64.89%)	23 (48.94%)	
**Current and previous smokers**	No	34 (36.17%)	15 (31.91%)	[1]0.330
	Yes	57 (60.64%)	32 (68.09%)	
	NA	3 (3.19%)	0 (0%)	-
**Most relevant comorbidities**	Hypertension	57 (60.64%)	27 (57.45%)	[1]0.720
	Cardiovascular disease	27 (28.72%)	14 (29.79%)	[1]0.843
	Asthma/chronic obstructive pulmonary disease	25 (26.6%)	13 (27.66%)	[1]0.552
	Diabetes mellitus	21 (22.34%)	6 (12.77%)	[1]0.182
	Previous thoracic surgery	8 (8.51%)	5 (10.64%)	[1]0.760
**Preoperative medications**	Anti-hypertensive agents	49 (52.13%)	26 (55.32%)	[1]0.858
	Rhythm/frequency control agents	38 (40.43%)	16 (34.04%)	[1]0.582
	Anticoagulants, antiaggregants	30 (31.91%)	14 (29.79%)	[1]0.849
	Other cardiovascular drugs (incl. diuretics)	8 (8.51%)	10 (21.28%)	[1]0.058
	Pulmonological drugs	21 (22.34%)	8 (17.02%)	[1]0.515
	Statins	16 (17.02%)	8 (17.02%)	[1]0.587
	Antidiabetics (incl. insulin)	17 (18.09%)	2 (4.26%)	[1]0.034
	Psychiatric drugs	19 (20.21%)	9 (19.15%)	[1]0.536
	Other medications	26 (27.66%)	8 (17.02%)	[1]0.211

[1] Fisher's exact test; [2] t-test; [3] Does not meet the criteria for Pearson's chi-squared test; [4] Pearson chi-squared. Normality was tested via visual interpretation (Q–Q plot). Continuous variables were tested via an independent samples t-test to compare differences between groups, whereas categorical variables were analyzed using Pearson's chi-squared test and Fisher's exact test to compare the proportions of groups.
